# BNIP3 (BCL2 interacting protein 3) regulates pluripotency by modulating mitochondrial homeostasis via mitophagy

**DOI:** 10.1038/s41419-022-04795-9

**Published:** 2022-04-11

**Authors:** Kun Liu, Qian Zhao, Hongyan Sun, Lei Liu, Chaoqun Wang, Zheng Li, Youqing Xu, Liang Wang, Lin Zhang, Honghai Zhang, Quan Chen, Tongbiao Zhao

**Affiliations:** 1 State Key Laboratory of Stem Cell and Reproductive Biology, Institute of Zoology, Chinese Academy of Sciences, 100101 Beijing, China; 2 Graduate University of Chinese Academy of Sciences, 100049 Beijing, China; 3 Beijing Institute for Stem Cell and Regenerative Medicine, 100101 Beijing, China; 4 School of Biological Sciences, Qufu Normal University, 273165 Qufu, China; 5 Department of Digestive System, Beijing Tiantan Hospital, Capital Medical University, 100070 Beijing, China

**Keywords:** Embryonic stem cells, Mitophagy

## Abstract

Autophagy-mediated mitochondrial degradation plays pivotal roles in both the acquisition and maintenance of pluripotency, but the molecular mechanisms that link autophagy-mediated mitochondrial homeostasis to pluripotency regulation are unclear. Here, we identified that the mitophagy receptor BNIP3 regulates pluripotency. In mouse ESCs, depletion of BNIP3 caused accumulation of aberrant mitochondria accompanied by decreased mitochondrial membrane potential, increased production of reactive oxygen species (ROS), and reduced ATP generation, which led to compromised self-renewal and differentiation. Impairment of mitophagy by knockdown of BNIP3 inhibited mitochondrial clearance during pluripotency induction, resulting in decreased reprogramming efficiency. These defects were rescued by reacquisition of wild-type but not LIR-deficient BNIP3 expression. Taken together, our findings highlight a critical role of BNIP3-mediated mitophagy in the induction and maintenance of pluripotency.

## Introduction

Autophagy was first identified as a non-selective degradation pathway triggered by starvation in somatic cells. Under nutrient deprivation, cells recycle their cytoplasmic contents by autophagic self-digestion to cope with the limited supplies of metabolites and energy [1, 2]. Nowadays, there is increasing evidence that basal autophagy serves as an intracellular quality control system by selectively degrading aggregated proteins and damaged organelles like mitochondria [3, 4]. Based on the discovery that an outer mitochondrial membrane protein, Uth1p, is required for efficient autophagic degradation of mitochondria in yeast, Dr. Lemasters proposed the term “mitophagy” to emphasize selective autophagy of the mitochondria [5, 6]. In yeast, ATG32 has been demonstrated as a mitochondrial receptor to regulate mitochondrial autophagy [7, 8].

Pluripotent stem cells (PSCs) include both embryonic stem cells (ESCs) and induced pluripotent stem cells (iPSCs). Compared with adult somatic cells, PSCs have fewer mitochondria and mainly rely on glycolysis rather than mitochondrial respiration for energy production. Mitochondria play vital roles in metabolism, apoptosis, Ca2+ signaling, the Krebs cycle, and oxidative phosphorylation. It is still controversial that whether mitochondria are involved in stemness regulation in PSCs. A previous study showed that mitochondrial respiration inhibition promoted pluripotency [9], while another report suggested that normal mitochondria only affected the proliferation of ESCs but not ESC pluripotency [10]. Recently, we identified that high autophagic flux, which regulate cellular protein and organelle homeostasis, was an intrinsic characteristic of mouse ESCs [11]. Loss of autophagy via deletion of Atg3 causes abnormal mitochondria accumulation in ESCs, leading to the breakdown of ESC self-renewal and pluripotency [12]. Consistently, studies have shown that activation of both canonical and noncanonical autophagy are essential for somatic cell reprogramming [13–15]. These findings support the view that mitochondrial autophagy is involved in pluripotency regulation. Here we screened the mitochondrial autophagy receptors in mouse ESCs to define whether and how these receptors regulate pluripotency.

## Results

### BNIP3 is essential for ESC self-renewal and pluripotency

To investigate the role of mitophagy in PSCs, we firstly used the 3-MA to inhibit the mitophagy in mouse ESCs. As expected, 3-MA treatments inhibited mitophagosome formation in ESCs (Supplementary Fig. S1A). As a consequence, the dysfunctional mitochondria accumulated in 3-MA treated ESCs (Supplementary Fig. S1B, C). Consistently, the self-renewal and pluripotency gene expression deteriorated in 3-MA treated ESCs (Supplementary Fig. S1D, E). These data indicated mitophagy play critical roles in PSCs.

**Fig. 1:**
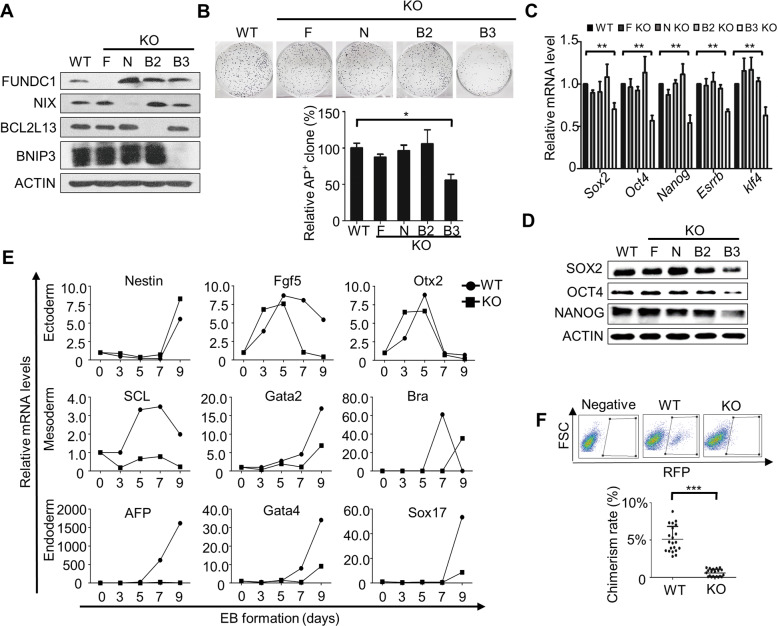
Knockout of Bnip3 impairs self-renewal and pluripotency of ESCs. **A** Proteins from wild-type (WT), Fundc1^−/−^ (F), Nix^−/−^ (N), Bcl2l13^−/−^ (B2), and Bnip3^−/−^ (B3) ESCs were detected by western blot. **B** Colony-formation assay of wild-type, Fundc1^−/−^, Nix^−/−^, Bcl2l13^−/−^, and Bnip3^−/−^ ESCs. Data are shown as mean ± SD, *n* = 3; *P < 0.05; Student’s *t* test. **C** Knockout of Bnip3 impairs ESC pluripotency. Real-time PCR analysis of the expression of pluripotency genes in WT and mitophagy receptor knockout ESCs. Data are shown as mean ± SD, **P < 0.01; Student’s *t* test. **D** Protein expression of SOX2, OCT4, and NANOG in wild-type and mitophagy receptor knockout ESCs. **E** Knockout of Bnip3 impairs lineage specification of ESCs. Real-time PCR analysis of the expression of lineage-specific genes in WT and Bnip3^−/−^ ESCs at the indicated days. Data shown are from one of three representative experiments. **F** The chimerism rate of WT and Bnip3^−/−^ ESCs. Embryos on 12.5 day were digested and analyzed by a FACS. Data are shown as mean ± SD, ***P < 0.001; Student’s *t* test.

In mammalian somatic cells, four outer mitochondrial membrane (OMM) proteins—FUNDC1, BCL2L13, NIX, and BNIP3—have been identified as receptors that directly interact with LC3 to mediate mitochondrial autophagy [16–19]. To explore whether these mitochondrial receptors function in mouse ESC identity maintenance, we established Fundc1, Bcl2l13, Nix and Bnip3 knockout ESC lines (Fig. 1A). The Bcl2l13 and Bnip3 knockout ESC lines were generated by CRISPR/Cas9-mediated gene editing (Supplementary Fig. S2A–D), while the Fundc1 and Nix null ESC lines were isolated from blastocysts of Fundc1 and Nix knockout mice. Colony-formation assays showed that knockout of Bnip3, but not Fundc1, Nix and Bcl2l13, significantly decreased the colony-formation ability of mouse ESCs, which indicates that BNIP3 is essential for ESC self-renewal (Fig. 1B). To test whether these four receptors are involved in pluripotency regulation, quantitative PCR and western blotting were employed to analyze the expression of pluripotency genes and their encoded proteins. Compared to Fundc1^−/−^, Nix^−/−^, and Bcl2l13^−/−^ ESCs, the Bnip3^−/−^ ESCs showed significantly decreased expression of pluripotency genes and proteins, which indicates that BNIP3 is pivotal for maintaining ESC pluripotency (Fig. 1C, D). In support of this observation, embryoid bodies (EBs) derived from Bnip3^−/−^ ESCs showed abnormal differentiation, characterized by delayed expression of certain endodermal and mesodermal marker genes (Fig. 1E).

We further employed the chimeric mouse formation assay to investigate the contribution of BNIP3 to differentiation in vivo. While both Bnip3^+/+^ and Bnip3^−/−^ ESCs formed chimeric mice, the chimeric mouse formation rate and the average chimerism rate of Bnip3^+/+^ ESCs were significantly higher than that of Bnip3^−/−^ ESCs, supporting the view that BNIP3 is critical for differentiation of pluripotent stem cells (Fig. 1F). Taken together, these data provide evidence that BNIP3 is essential for ESC self-renewal and regulates ESC pluripotency and differentiation.

### BNIP3 maintains mitochondrial homeostasis in mouse ESCs

**Fig. 2:**
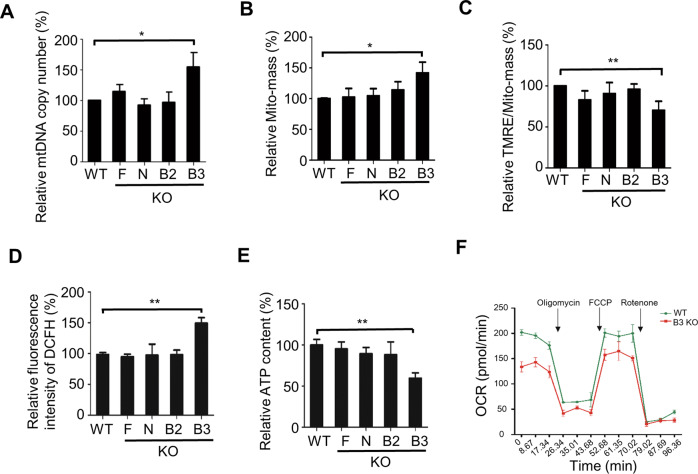
BNIP3 protects mitochondrial homeostasis in mouse ESCs. **A** Real-time PCR analysis of mtDNA in WT and mitophagy receptor knockout ESCs. Data are shown as mean ± SD, *n* = 3; *P < 0.05; Student’s *t* test. **B** Analysis of mitochondrial mass (Mito-mass) in WT and mitophagy receptor knockout ESCs. MitoTracker Green was used for Mito-mass detection. Data are shown as mean ± SD, *n* = 3; *P < 0.05; Student’s *t* test. **C** Mitochondrial membrane potential of WT and mitophagy receptor knockout ESCs. Data are shown as mean ± SD, *n* = 3; **P < 0.01; Student’s *t* test. **D** ROS generation in WT and mitophagy receptor knockout ESCs. Data are shown as mean ± SD, *n* = 3; **P < 0.01; Student’s *t* test. **E** ATP generation in WT and mitophagy receptor knockout ESCs. Data are shown as mean ± SD, *n* = 3; **P < 0.01; Student’s *t* test. **F** Oxygen consumption rates (OCRs) of WT and Bnip3^−/−^ ESCs treated sequentially with oligomycin, FCCP, and rotenone.

We then aimed to decipher the mechanisms by which mitophagy regulates pluripotency. To investigate whether the mitophagy receptors FUNDC1, BCL2L13, NIX, and BNIP3 are involved in mitochondrial homeostasis regulation in ESCs, we evaluated mitochondrial quantity and function in Fundc1^−/−^, Bcl2l13^−/−^, Nix^−/−^, and Bnip3^−/−^ ESCs. A quantitative polymerase chain reaction (PCR) assay using mitochondrial DNA (mtDNA) as a template was performed to track differences in the mtDNA copy number between these cells. Interestingly, the number of mtDNA copies in Bnip3^−/−^ ESCs, but not Fundc1^−/−^, Nix^−/−^, and Bcl2l13^−/−^ ESCs, is significantly higher than that in wild-type (WT) ESCs (Fig. 2A). This indicates that BNIP3 contributes to constitutive mitochondrial removal in ESCs under normal conditions. Consistent with these observations, Bnip3 knockout leads to an increase in the total mitochondrial mass in cells (Fig. 2B).

We next investigated whether the lack of BNIP3 affects mitochondrial function. In contrast to the knockout of Fundc1, Nix, and Bcl2l13, silencing of Bnip3 significantly decreased the mitochondrial membrane potential, oxygen consumption and ATP production in ESCs, without influencing glycolysis rate (Fig. 2C, E, F and Supplementary Fig. S3C). At the same time, ROS production was significantly higher in Bnip3^−/−^ than in Bnip3^+/+^ ESCs (Fig. 2D).

In addition, we tested the expression of mitochondrial fission-related proteins and found no expression differences in BNIP3^+/+^ and Bnip3^−/−^ ESCs (Supplementary Fig. S3A, B). It has been reported that phosphorylation of Drp1 at S616 by Pink1 induces mitophagy-independent mitochondrial fission in somatic HEK293 cells [20], and Redox oxidative species (ROS)-mediated phosphorylation of Drp1^S616^ promotes mitochondrial fission in triple-negative breast cancer cells [21]. In consistence, enhanced phosphorylation of Drp1 at S616 was found in Bnip3 knockout ESCs compared to WT ESCs, indicating the existence of mitophagy-independent mitochondrial fission that might be induced by ROS in Bnip3^−/−^ ESCs (Supplementary Fig. S3B).

Taken together, these data support the view that BNIP3 is required for maintaining the numbers and functional integrity of mitochondrion, and is therefore important for regulating mitochondrial homeostasis.

### BNIP3-mediated mitophagy function is required for mitochondrial homeostasis and guards ESC identity

**Fig. 3:**
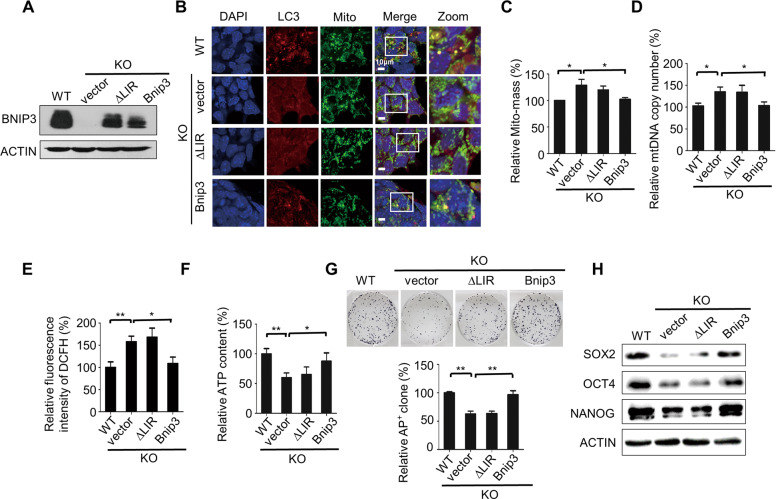
BNIP3-mediated mitophagy guards ESC identity. **A** Characterization of ESCs for Bnip3 rescue experiments. Western blot detection of the expression of Bnip3 in WT ESCs, control Bnip3^−/−^ ESCs (vector), Bnip3^−/−^ ESCs expressing the Bnip3 ∆LIR mutant, and Bnip3^−/−^ ESCs expressing WT Bnip3. **B** Mitophagosomes in Bnip3^+/+^, Bnip3^−/−^, and Bnip3 rescued ESCs. Upon mitophagy occurs, abnormal mitochondria are sequestrated into autophagosomes and subsequent traffic to lysosomes for degradation. The colocalization of mitochondrial probes (MitoTracker Green) with markers of the autophagic machinery (LC3) indicates mitophagosome. ESCs were stained by MitoTracker Green for 30 min and then stained by autophagic machinery maker LC3. Colocalization of two fluorescent proteins were observed by using fluorescence microscopy. Blue: DAPI; red: LC3; green: MitoTracker Green. **C** Increased Mito-mass in Bnip3^−/−^ ESCs is rescued by the gain of expression of WT but not LIR-deficient Bnip3. Data are shown as mean ± SD, *n* = 3; *P < 0.05; Student’s *t* test. **D** Increased mtDNA copy number in Bnip3^−/−^ ESCs is rescued by the gain of expression of WT but not LIR-deficient Bnip3. Data are shown as mean ± SD, *n* = 3; *P < 0.05; Student’s *t* test. **E** Enhanced ROS generation in Bnip3^−/−^ ESCs is rescued by the gain of expression of WT but not LIR-deficient Bnip3. Data shown as mean ± SD, *n* = 3; *P < 0.05; **P < 0.01; Student’s *t* test. **F** Defective ATP generation in Bnip3^−/−^ ESCs is rescued by the gain of expression of Bnip3^+/+^ but not Bnip3^△LIR^. Data are shown as mean ± SD, *n* = 3; *P < 0.05; **P < 0.01; Student’s *t* test. **G** Aberrant self-renewal of Bnip3^−/−^ ESCs, as judged by colony-formation assays, is rescued by the gain of expression of Bnip3^+/+^ but not Bnip3^△LIR^. Data are shown as mean ± SD, *n* = 3; **P < 0.01; Student’s *t* test. **H** Western blot of SOX2, OCT4, and NANOG in WT, Bnip3^−/−^, and Bnip3-rescued ESCs.

The BH3-only protein BNIP3 is primarily localized to the mitochondria and contains a LIR (Microtubule-associated Protein 1 Light Chain 3 (LC3) interacting region) that is required for autophagic removal of mitochondria in somatic cells. Single amino acid mutations in the LIR motif of BNIP3 significantly reduce mitochondrial autophagy but do not alter its pro-death activity [17, 22, 23]. To test whether the abnormal accumulation of mitochondria and aberrant self-renewal ability of Bnip3^−/−^ ESCs was directly caused by the loss of BNIP3-mediated mitophagy, gain-of-function assays were performed by introducing Bnip3 expression into Bnip3^−/−^ ESCs. We established stable Bnip3^−/−^ ESC lines carrying an empty vector, wild-type Bnip3, and a LIR-deficient mutant (Bnip3^△LIR^) of Bnip3 (Fig. 3A). We found that ectopic expression of wild-type Bnip3 but not Bnip3^△LIR^ in Bnip3^−/−^ ESCs rescued the decreased number of mitophagosomes (Fig. 3B). In addition, the increased mitochondrial mass and mtDNA copy number in Bnip3^−/−^ ESCs were significantly reduced by the gain of wild-type Bnip3 expression (Fig. 3C, D), which indicates that the accumulation of abnormal mitochondria in Bnip3-deficient ESCs was directly caused by defects in BNIP3-dependent mitophagy. Furthermore, ROS production and ATP generation were also rescued by the reacquisition of wild-type Bnip3 but not the LIR-deficient mutant Bnip3^△LIR^ (Fig. 3E, F). Accordingly, the abnormal self-renewal and compromised expression of pluripotency genes in Bnip3-deficient ESCs were restored by reintroducing wild-type Bnip3 but not the LIR-deficient mutant (Fig. 3G, H). Also, knockout of Bnip3 did not affect proliferation and apoptosis of ESCs (data not shown). Together, these data strongly suggest that BNIP3-dependent mitophagy maintains ESC mitochondrial homeostasis and guards ESC identity.

### Mitochondria are cleared by autophagy during somatic cell reprogramming

We next asked whether mitochondrial autophagy contributes to mitochondrial remodeling during somatic cell reprogramming. To this end, we first assessed changes in mitochondrial mass by MitoTracker staining of cells undergoing reprogramming (Supplementary Fig. S4A, B). As expected, the total mitochondrial mass was gradually reduced as reprogramming progressed (Supplementary Fig. S4B, C), which indicates that mitochondria were removed during the reprogramming process. We then determined whether autophagy is responsible for mitochondrial remodeling during reprogramming. On reprogramming day 2, the colocalization of LC3 with mitochondria and the number of acidic puncta in cells were significantly increased (Supplementary Fig. S4D, E). This indicates that the mitochondria were actively delivered to autophagosomes and degraded by autophagy upon initiation of reprogramming. In support of these observations, transmission electronic microscopy detected autophagosomes containing mitochondria in cells at reprogramming day 2 (Supplementary Fig. S4F). The established iPSCs have a normal karyotype, express pluripotent markers and differentiate normally, all of which are indicators of successful reprogramming (Supplementary Fig. S5A–F). Together these data provide evidence that mitochondria are degraded by autophagy during reprogramming.

### BNIP3 regulates mitochondrial removal and somatic cell reprogramming

To determine how mitochondria are degraded by autophagy during somatic cell reprogramming, we isolated wild-type, Nix^−/−^ knockout and Fundc1^−/−^ knockout MEFs, and designed specific shRNAs targeting Bcl2l13 and Bnip3 (Supplementary Fig. S6A, C, E, G). Complete inactivation of Nix or Fundc1 did not affect reprogramming efficiency, indicating that neither NIX nor FUNDC1 are involved in somatic cell reprogramming (Supplementary Fig. S6B, D). While knockdown of Bcl2l13 did not impair the reprogramming efficiency, Bnip3 knockdown significantly decreased the reprogramming efficiency (Supplementary Fig. S6F, H). This indicates that BNIP3 is not only essential for pluripotency maintenance but is also crucial for pluripotency induction.

**Fig. 4:**
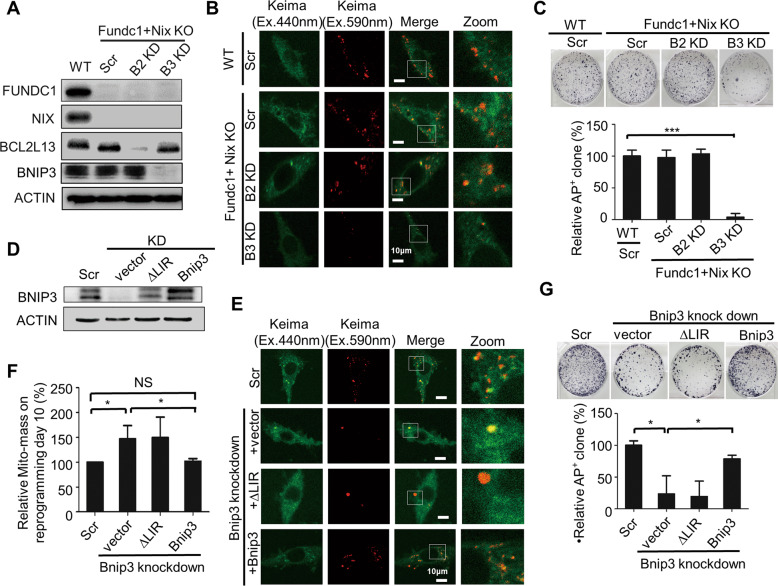
BNIP3-dependent mitophagy is essential for reprogramming. **A** Western blot of WT, Nix^−/−^/Fundc1^−/−^, Nix^−/−^/Fundc1^−/−^/Bcl2l13^KD^, and Nix^−/−^/Fundc1^−/−^/Bnip3^KD^ MEFs. **B** Knockdown of Bnip3 expression in Nix^−/−^/Fundc1^−/−^ MEFs inhibits mitophagy during reprogramming. Keima (Ex.440 nm): mitochondria in a neutral pH environment; (Ex.590 nm): mitochondria in an acidic pH environment. **C** Alkaline phosphatase (AP) staining of iPSCs on reprogramming day12. Data are shown as mean ± SD, *n* = 3; ***P < 0.001; Student’s *t* test. **D** Western blot to detect expression of Bnip3 in Bnip3 knockdown MEFs. **E** Mitophagy is rescued by the gain of expression of Bnip3^+/+^ but not Bnip3^△LIR^ in Bnip3 knockdown MEFs. Keima (Ex.440 nm): mitochondria in a neutral pH environment; Keima (Ex.590 nm): mitochondria in an acidic pH environment. **F** Increased Mito-mass in Bnip3 knockdown cells at reprogramming day 10 is rescued by the gain of expression of Bnip3^+/+^ but not Bnip3^△LIR^. Data are shown as mean ± SD, *n* = 3; *P < 0.05; Student’s *t* test. **G** Restoration of reprogramming efficiency by the gain of expression of Bnip3^+/+^ but not Bnip3^△LIR^. Data are shown as mean ± SD, *n* = 3; *P < 0.05; Student’s *t* test.

To further confirm that BNIP3 is specifically required for somatic reprogramming, we isolated Nix^−/−^/Fundc1^−/−^ double knockout MEFs, and knocked down the expression of either Bcl2l13 or Bnip3 in these cells before assessing the mitochondrial changes and reprogramming efficiency (Fig. 4A). On reprogramming day 2, the mitophagosome number was decreased in Nix^−/−^/Fundc1^−/−^/Bnip3^KD^ cells, but not in Nix^−/−^/Fundc1^−/−^ cells or Nix^−/−^/Fundc1^−/−^/Bcl2l13^KD^ cells (Fig. 4B). This indicates that BNIP3 specifically regulates mitochondrial clearance during reprogramming. Correspondingly, the reprogramming efficiency was significantly inhibited in Nix^−/−^/Fundc1^−/−^/Bnip3^KD^ cells, but not in Nix^−/−^/Fundc1^−/−^ cells or Nix^−/−^/Fundc1^−/−^/Bcl2l13^KD^ cells (Fig. 4C).

### BNIP3-dependent mitophagy function is essential for somatic cell reprogramming

To further confirm that impaired reprogramming of Bnip3^−/−^ MEFs results from a defect in BNIP3-mediated mitochondrial autophagy, gain-of-function assays were performed by overexpressing both wild-type Bnip3 and LIR-deficient mutant Bnip3^△LIR^ in Bnip3^−/−^ MEFs (Fig. 4D). We found that ectopic expression of wild-type Bnip3 but not LIR-deficient mutant Bnip3^△LIR^ in Bnip3^−/−^ MEFs rescued both the defective mitochondrial clearance and the reduced reprogramming efficiency (Fig. 4E–G). In addition, we established iPSC lines with decreased Bnip3 expression (Supplementary Fig. S7A), and found inhibition of Bnip3 resulted in abnormal mitochondrial homeostasis, pluripotency gene expression, and teratoma formation (Supplementary Fig. S7B–G). These results support the view that BNIP3-dependent mitophagy is essential for mitochondrial remodeling and somatic cell reprogramming.

## Discussion

Based on the fact that the mitochondrion is the organelle that generates ATP by oxidative phosphorylation, and somatic cells have more mitochondria than pluripotent stem cells (PSCs), it is widely accepted that somatic cells rely heavily on oxidative phosphorylation while pluripotent stem cells favor glycolysis for energy production [24, 25]. Accordingly, it has been observed that a transition from somatic mitochondrial oxidative metabolism to glycolysis is required for successful reprogramming [26]. These results indicate that mitochondria must be remodeled during the reprogramming of somatic cells and that PSCs must employ unique molecular mechanisms to regulate mitochondrial homeostasis. In support of this view, recent studies have shown that both canonical and noncanonical autophagy mediate mitochondrial remodeling during somatic cell reprogramming [12, 14]. Furthermore, high autophagic flux has been identified as an intrinsic characteristic of ESCs to maintain mitochondrial homeostasis and pluripotency [11]. These data suggest that mitochondria are precisely regulated by autophagy during the induction and maintenance of pluripotency. However, the mechanisms by which PSCs harness autophagy to regulate mitochondrial homeostasis have not been clearly defined, nor is it fully understood how autophagy regulates mitochondrial remodeling during somatic reprogramming. Here we identified that the BCL2 interacting protein 3 is the mitophagy receptor that regulates mitochondrial homeostasis in ESCs and contributes to mitochondrial remodeling during somatic cell reprogramming.

To date, four outer mitochondrial membrane (OMM)-anchored proteins—FUNDC1, BCL2L13, NIX, and BNIP3—have been identified to serve as direct receptors for mitochondrial autophagy. Through their conserved LIR motifs, they link with LC3 for autophagic degradation of mitochondria in mammalian cells. These autophagy receptors are required for multiple mitophagy programs that operate either independently or are linked by crosstalk [27]. FUNDC1 is a mitochondrial receptor for hypoxia-induced mitophagy, which can be negatively regulated by Src kinase and casein kinase 2 [18]. BCL2L13 is a newly identified mitophagy receptor in mammalian somatic cells, which can induce mitochondrial fragmentation and Parkin-independent mitophagy [19]. BNIP3 and its homolog NIX are atypical members of the pro-apoptotic Bcl-2 subfamily, which can not only promote cellular apoptosis via their BH3 domains but can also induce mitophagy via their LIR motifs [27]. While NIX was demonstrated to be required for mitochondrial clearance during reticulocyte development, BNIP3 was identified to mediate mitochondrial autophagy in cardiomyocytes and liver cells [22, 28]. By screening these OMM-anchored proteins, we identified that mitophagy mediated by BNIP3, but not the other mitochondrial receptors, is required for both maintenance and acquisition of pluripotency. In contrast, a recent study has defined that mitochondrial autophagy mediated by NIX but not BNIP3 is critical for three factor-induced reprogramming (Oct4, Sox2, and Klf4) (Xiang et al., 2017). The same study also found that NIX is not involved in four factor-induced reprogramming (Oct4, Sox2, Klf4, and cMyc), which is consistent with our results [29]. However, the mechanisms that underlie the functional divergence between NIX and BNIP3 in pluripotency regulation need further investigation.

The OMM proteins p62, NBR1, and OPTN serve as adaptors that link signals from depolarized mitochondria, which are sensed by PINK1/PARK2, with autophagic signals to trigger mitochondrial autophagy [27]. The fact that Park2 depletion does not affect mitochondrial clearance and reprogramming efficiency supports the view that PINK1/PARK2-mediated selective mitophagy is not involved in pluripotency regulation [12]. To further clarify this point, we investigated the contribution of mitochondrial depolarization to somatic reprogramming and pluripotency. We found that FCCP treatment, which enhances mitochondrial depolarization, did not increase reprogramming efficiency or promote pluripotency. In agreement with these observations, the recent study of Xiang et al. showed that mitochondria with both high and low membrane potential were delivered to autophagosomes for degradation during reprogramming, supporting the view that somatic cell reprogramming is independent of mitochondrial depolarization [29]. We propose that the autophagic regulation of mitochondrial homeostasis in PSCs does not occur through recognition of damaged mitochondria, but instead involves the active degradation of mitochondria using a mitophagy receptor by undefined mechanisms.

In conclusion, we demonstrated that the BNIP3-mediated mitophagy pathway is critical for both maintenance and acquisition of pluripotency. This study gives new insights into our current understating of the metabolic regulation of pluripotency and provides potential novel targets for the manipulation of stem cell fate.

## Materials and methods

### Animals

GFP-LC3 mice (RBRC00806) [30] and Mito-RFP mice (RBRC03743) [31] were purchased from Riken BioResource Centre. Dr. Quan Chen provided Fundc1 knockout mice and Fundc1&Nix double knockout mice. Nix knockout mice were provided under permission by Dr. Paul A Ney. All protocols used for animal manipulation were approved by the Institutional Animal Care Committee.

### Reagents

TMRE (22220) was purchased from AAT Bioquest. MitoTracker Green (40742ES50) and MitoTracker Red (40743ES50) were purchased from Yeasen. FCCP (C2920) and CFSE (21888) were purchased from Sigma Aldrich.

### Antibodies

The following antibodies were used: anti-Fundc1 polyclonal antibody (P050, AVIVA), anti-Nix polyclonal antibody (CST12396, Cell Signaling Technology), anti-Bcl2L13 polyclonal antibody (16612, Proteintech), anti-Bnip3 polyclonal antibody (#3769, Cell Signaling Technology), anti-Drp1 monoclonal antibody (611113, BD), anti-Mff polyclonal antibody (17090-1-AP, Proteintech), anti-Fis1 polyclonal antibody (10956-1-AP, Proteintech), anti-Actin monoclonal antibody (A5441, Sigma Aldrich), anti-LC3B antibody (PM036, Medical and Biological Laboratories Co.), anti-p-Drp1(S616) antibody (#3455, Cell Signaling Technology), anti-Tim23 antibody (FNab08693, FineTest), Anti-LC3A/B antibody for immunofluorescence (ab128025, Abcam), donkey anti-rabbit IgG (H + L) highly cross-adsorbed secondary antibody, Alexa Fluor 594 (A21207, Invitrogen Thermo Fisher Scientific), Alexa Fluor 488 donkey anti-rabbit IgG (A21206, Invitrogen Thermo Fisher Scientific), SSEA-1 (SC-21702AF647, Santa Cruz Biotechnology).

### Plasmids

pMXs-Oct4(13366), pMXs-Sox2(13367), pMXs-Klf4(13370), and pMXs-cMyc(13375) were purchased from Addgene. Fundc1, Nix, Bcl2L13, Bnip3, and the Bnip3 LIR mutant were cloned into pMXs and pCDH-CAG-RFP lentivectors as described previously.

### ESC isolation and iPSC induction

Fundc1 knockout, Nix knockout, Fundc1&Nix double knockout, and wild-type ESCs were isolated at day E3.5 and seeded on feeder layers for selection using 2i medium. Then ESCs were cultured for three to five passages and maintained in ESC medium [12]. For iPSC induction, 50,000 MEF cells/well were seeded in a six-well plate and followed by infection with four reprogramming retrovirus cocktail; iPSC colonies were picked 14 days after infection as described previously [12].

### Generation of Bcl2L13 knockout and Bnip3 knockout ESCs

Bcl2L13 knockout ESCs and Bnip3 knockout ESCs were generated using the CRISPR/Cas9 system. Bcl2L13 and Bnip3 gRNA were cloned into px330 vector, and transfected into ESCs with a Gene Pulser Xcell II (Bio-Rad) according to the manufacturer’s protocols. Then knockout ESCs were identified by T7E1 enzyme and sequencing. All knockout ESCs were tested by western blotting.

### Western blotting

Cells were lysed in RIPA buffer (50 mM Tris-HCl, pH 7.4, 150 mM NaCl, 0.5% sodium deoxycholate, 1% Nonidet P-40, 5 mM EGTA, 2 mM EDTA, 10 mM NaF) with protease inhibitor cocktail (04693116001, Roche). Equivalent protein quantities (20 µg) were subjected to SDS-PAGE, and transferred to nitrocellulose membranes (Millipore). Then the membranes were probed with the indicated primary antibodies, followed by the appropriate HRP-conjugated secondary antibodies (Santa Cruz). Immunoreactive bands were detected with a Luminata Forte Western HRP Substrate Kit (WBLUF0100, Millipore).

### Determination of mtDNA copy number and mitochondrial mass

A TIANamp Genomic DNA Kit (Tiangen Biotech [Beijing] Co., DP304-03) was used for DNA extraction. Quantitative real-time PCR was used for mtDNA copy number detection and genomic DNA was loaded as control. The primers used were as previously described [32]. MitoTracker Green (100 nM) was used for mitochondrial mass detection, and the cells were stained at 37 °C for 30 min, then analyzed on a FACS-Calibur flow cytometer.

### Fluorescence microscopy

To detect LC3, MEFs/ESCs were transfected with LC3-GFP vector. For detection of Mito-RFP staining, cells were treated with MitoTracker for 30 min at 37 °C. For immunostaining of Bnip3, cells were cultured on gelatin-coated glass slides, fixed in 4% PFA for 20 min, washed with DPBS, permeabilized by 0.2% Triton-X100 for 0.5 h, blocked by 2% BSA for 1 h, stained with the appropriate primary antibody overnight at 4 °C, and then incubated with secondary antibody for 2 h at 37 °C. Cell nuclei were stained with DAPI. For detection of Mito-Keima, ESCs/MEFs were transfected with a plasmid expressing Mito-Keima and directly monitored by confocal microscopy.

### Transmission electron microscopy

Cells were fixed in 2.5% glutaraldehyde for 2 h at 4 °C, then immersed in 2% osmium tetroxide. After fixation, the sample was dehydrated sequentially in 50, 70, 90, 95, and 100% ethanol. Copper grids were used to collect ultrathin sections, and uranyl acetate and lead citrate were used for counterstaining. Images were taken with a FEI Tecnai spirit transmission electron microscope.

### Quantitative real-time PCR

Total RNA was extracted from samples with an RNeasy Total RNA Isolation Kit (Qiagen, 74104). Then total RNA was reverse transcribed into cDNA using a SuperScript^TM^ III First-Strand Synthesis System (Invitrogen Thermo Fisher Scientific, 18080051) according to the manufacturer’s instructions. The primers used are the same as previously published [12, 33].

### Measurement of OCR, ROS, and ATP

Seahorse XF24 analyzer (Seahorse Bioscience Asia, Shanghai) was used for measuring respiration in intact cells. Briefly, ESCs were seeded at 60,000 cells/well for 6 h, and measurements were made in strict accordance with the standard protocol in the manual. A CellTiter-GloLuminescent Cell Viability Assay kit (Promega Corporation, 0000092970) was used to measure cellular ATP content. Cellular ROS was measured by flow cytometry using HDCF-DA from Sigma Aldrich (D6883).

### AP staining and colony-formation assay

AP staining was performed with a BCIP/NBT Alkaline Phosphatase Colour Development Kit (Beyotime) according to the manufacturer’s instructions. The colony-formation assay was used as described [12]. Briefly, ESCs were trypsinized into single cells, seeded at 1000 cells per well into a 6-well plate (coated with feeder cells), and cultured for a week. After AP staining, the number of AP-positive colonies was counted.

## Supplementary information

Supplemental Figure legends

Supplementary Figure 1

Supplementary Figure 2

Supplementary Figure 3

Supplementary Figure 4

Supplementary Figure 5

Supplementary Figure 6

Supplementary Figure 7

checklist

## Data Availability

The data and materials during this study are available from the corresponding author on reasonable request.
