# Supplementary material for: BNIP3 (BCL2 interacting protein 3) regulates pluripotency by modulating mitochondrial homeostasis via mitophagy
Source: Cell Death Dis. 2022 Apr 11;13(4):334. doi: 10.1038/s41419-022-04795-9 (PMC9001722; doi:10.1038/s41419-022-04795-9)
Supplement: Supplementary file 1 — Supplemental Figure legends [file 41419_2022_4795_MOESM1_ESM.docx]

**Supplementary Figure 1.** Inhibition of mitophagy by 3-MA impaired mitochondrial homeostasis and ESC identity. (**A**) 3-MA inhibited mitophagosome formation in ESCs. LC3-GFP, Mito-RFP ESCs were treated with DMSO or 3-MA (5 mM) for 4h, then fixed and stained with DAPI for imaging. (**B**) Inhibition of mitophagy by 3-MA increased Mito-mass of ESCs. ESCs were treated with DMSO or 3-MA, and stained with Mito-tracker green for FACS analysis. Data are shown as mean ± SD, n=3; *, P<0.05; Student’s t-test. (**C**) Inhibition of mitophagy by 3-MA decreased relative mitochondrial membrane potential of ESCs. Data are shown as mean ± SD, n=3; *, P<0.05; Student’s t-test. (**D**) 3-MA impaired self-renewal of ESCs. Data are shown as mean ± SD, n=3; **, P<0.01; Student’s t-test. (**E**) 3-MA inhibited pluripotency gene expression of ESCs. Data are shown as mean ± SD, n=3; *, P<0.05; Student’s t-test.

**Supplementary Figure 2.** Generation of Bcl2l13 and Bnip3 knockout ESCs by CRISPR-Cas9 system. (**A**) Schematic of Cas/sgRNA-targeting sites in Bcl2l13 genome loci. (**B**) Western blot to identify the knockout of Bcl2l13. (**C**) Schematic of Cas/sgRNA-targeting sites in Bnip3 genome loci. (**D**) Western blot to identify the knockout of Bnip3.

**Supplementary Figure 3.** Deletion of Bnip3 did not affect mitochondrial fission and glycolysis in ESCs. (**A**) Real-time PCR of mitochondrial fission related genes in WT and Bnip3^-/-^ ESCs. (**B**) Western blot of mitochondrial fission related gene expression in WT and Bnip3^-/-^ ESCs. Quantifications of each protein expression were shown (*P < 0.05, n = 3) The loading control was TIM23. (**C**) Extracellular acidification rate (ECAR) of WT and Bnip3^-/-^ ESCs treated sequentially with glucose, oligomycin and 2-DG.

**Supplementary Figure 4.** Somatic cell reprogramming induces mitochondrial autophagy. (**A**) Schematic diagram of the somatic cell reprogramming protocol. (**B**) Mito-mass gradually decreased during reprograming. Ctrl; cells without reprograming; R cell: cells undergoing reprogramming; Fluorescence of Mito-RFP cells was detected at indicated reprogramming days. (**C**) Fluorescence intensity of mitochondria in reprograming. The data were record of (**B**). (**D**) Reprogramming induced mitochondrial autophagy. Blue: DAPI; Red: Mitochondria; Green: LC3. Mito-RFP LC3-GFP MEF were used for monitoring mitochondrial autophagy at reprogramming day 2. Co-localizations of mitochondria and LC3 were counted in 50 cells. Data shown as mean ± SD, n=3; **, P<0.01; Student’s t-test. (**E**) Monitoring mitochondrial autophagy during reprogramming by mito-Keima system. Keima(Ex.440nm): mitochondria in neutral pH environment; Keima(Ex.590nm): mitochondria in acidic pH environment; the acidic puncta were counted in 50 cells. Data shown as mean ± SD, n=3; **, P<0.01; Student’s t-test. (**F**) The representative transmission electronic microscopy pictures of autophagic mitochondria at reprogramming day 2.

**Supplementary Figure 5.** Characterization of iPSCs. (A) Phase‐contrast microphotograph image of cells on reprogramming day 0, day 4,day 8 and day 12. (**B**) Karyotype of iPSCs. The number of chromosome is 40. (**C**) iPSCs are SSEA-1 positive. (**D**) Pluripotent gene expression of iPSCs are similar to ESCs. (**E**) HE staining on iPSC formed teratoma. (**F**) Chimera mice generated from iPSCs.

**Supplementary Figure 6.** Screening of mitophagy receptor required for reprogramming. (**A**) Western blot of WT and Fundc1 KO MEF. (**B**) Deletion of Fundc1 did not affect reprogramming. NS, no significant difference. (**C**) Western blot of WT and Nix KO MEF. (**D**) Deletion of Nix does not affect reprogramming. NS, no significant difference. (**E**) Western blot of Bcl2l13 knockdown MEF. (**F**) Knockdown of Bcl2l13 did not affect reprogramming. NS, no significant difference. (**G**) Western blot of Bnip3 knockdown MEF. (**H**) Knockdown of Bnip3 significantly decreased reprogramming efficiency. Data shown as mean ± SD, n=3; ***, P<0.001; Student’s t-test.

**Supplementary Figure 7.** Knockdown of Bnip3 causes dysfunctional mitochondrial accumulation leading to defective iPSC identity. (**A**) Real-time PCR of Bnip3 in Scramble and Bnip3 knockdown iPSCs. Data are shown as mean ± SD, n=3; **, P<0.01; Student’s t-test. (**B**) Knockdown of Bnip3 during reprogramming results in increased Mito-mass in established iPSCs. Data are shown as mean ± SD, n=3; **, P<0.01; Student’s t-test. (**C**) Knockdown of Bnip3 during reprogramming results in decreased mitochondrial membrane potential in established iPSCs. Data are shown as mean ± SD, n=3; *, P<0.05; Student’s t-test. (**D**) Enhanced ROS was observed in Bnip3 knockdown iPSCs. Data are shown as mean ± SD, n=3; *, P<0.05; Student’s t-test. (**E**) Decreased ATP content was observed in Bnip3 knockdown iPSCs. Data are shown as mean ± SD, n=3; **, P<0.01; Student’s t-test. (**F**) Pluripotency gene expression in Scramble and Bnip3 knockdown iPSCs. Data are shown as mean ± SD, n=3; *, P<0.05; Student’s t-test. (**G**) Teratoma from Scramble and Bnip3 knockdown iPSCs.
