# Supplementary figures and images for: BNIP3 (BCL2 interacting protein 3) regulates pluripotency by modulating mitochondrial homeostasis via mitophagy
Source: Cell Death Dis. 2022 Apr 11;13(4):334. doi: 10.1038/s41419-022-04795-9 (PMC9001722; doi:10.1038/s41419-022-04795-9)

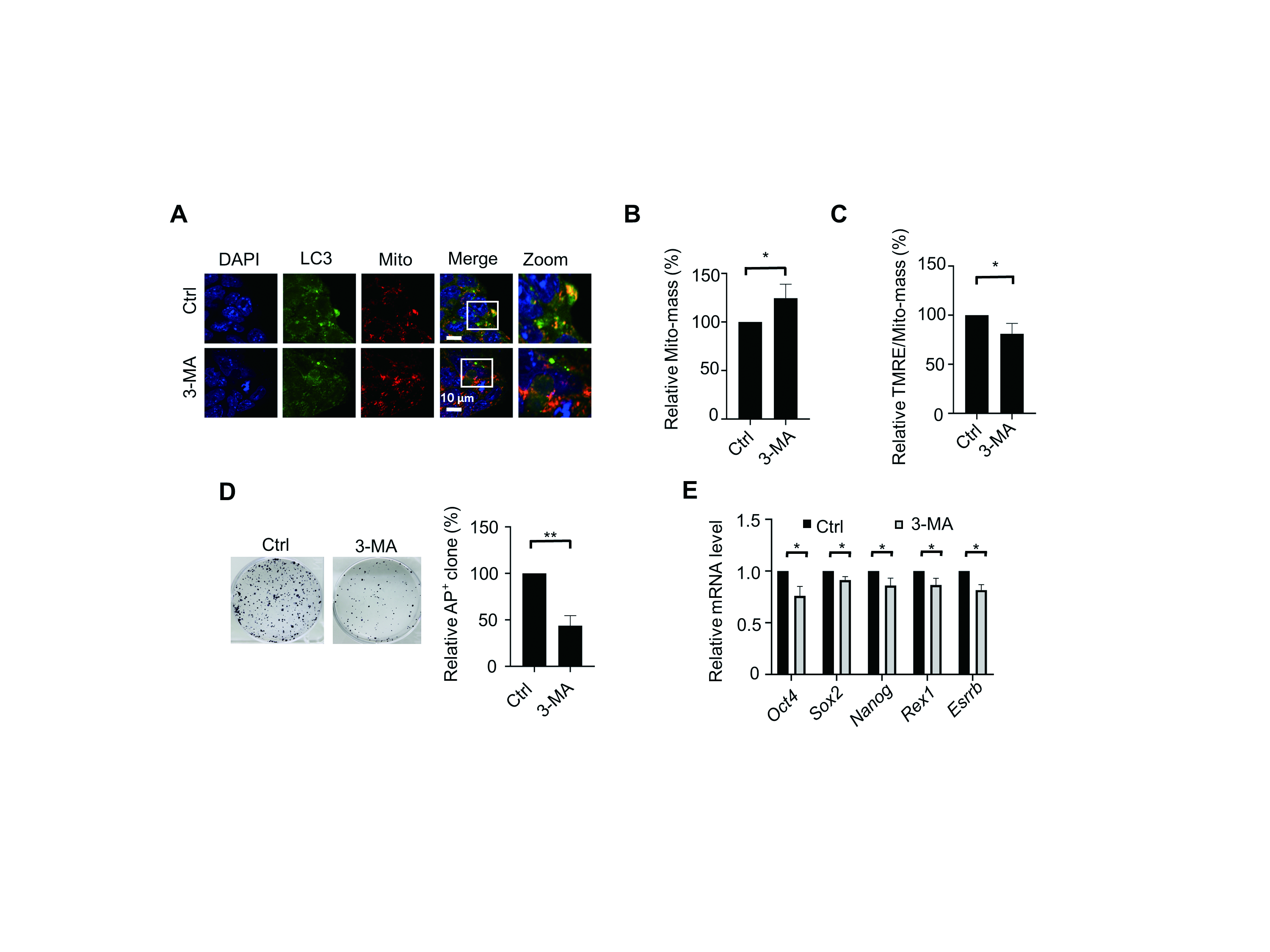

Supplement: Supplementary file 2 — Supplementary Figure 1 [file 41419_2022_4795_MOESM2_ESM.tif]

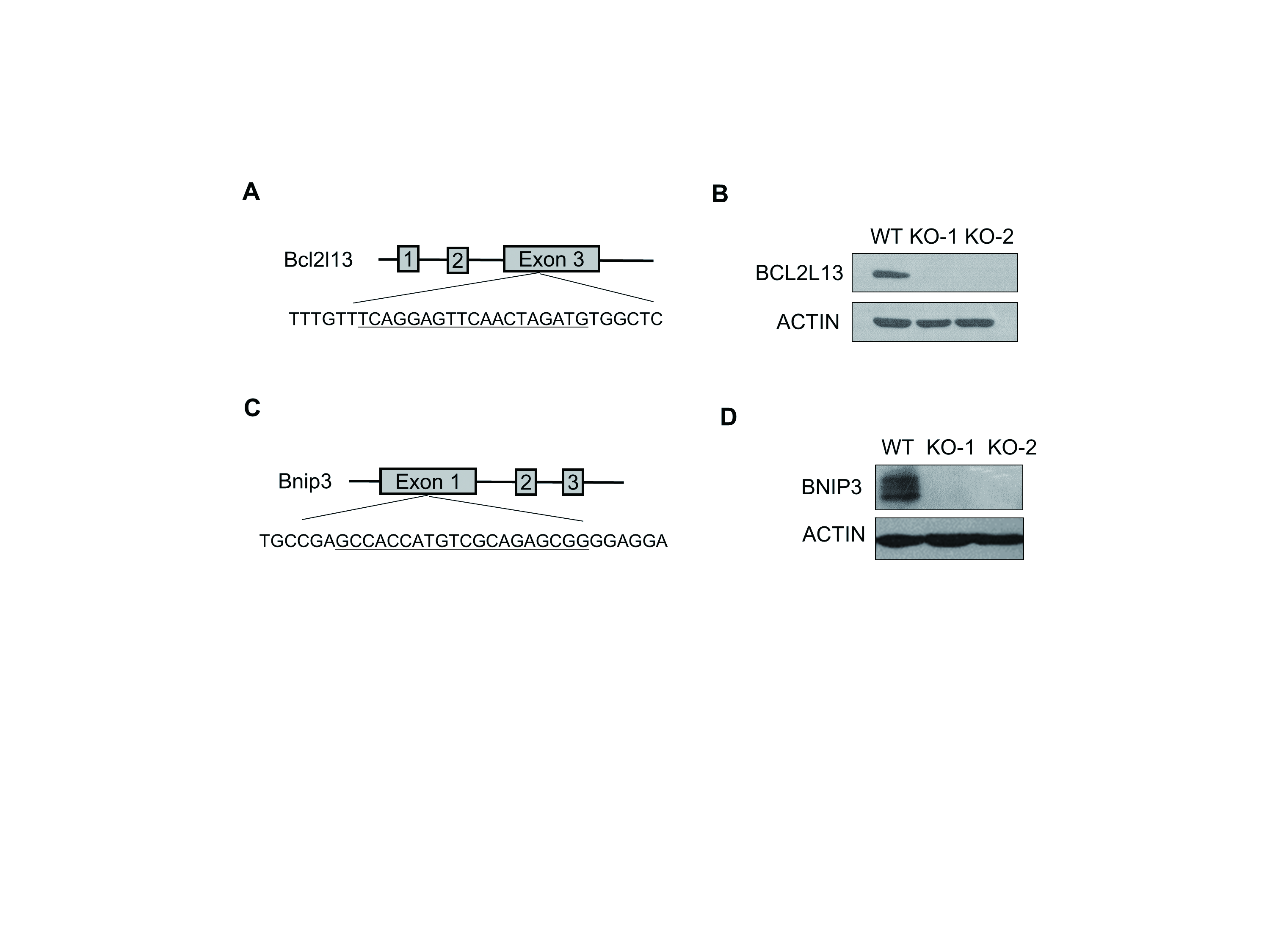

Supplement: Supplementary file 3 — Supplementary Figure 2 [file 41419_2022_4795_MOESM3_ESM.tif]

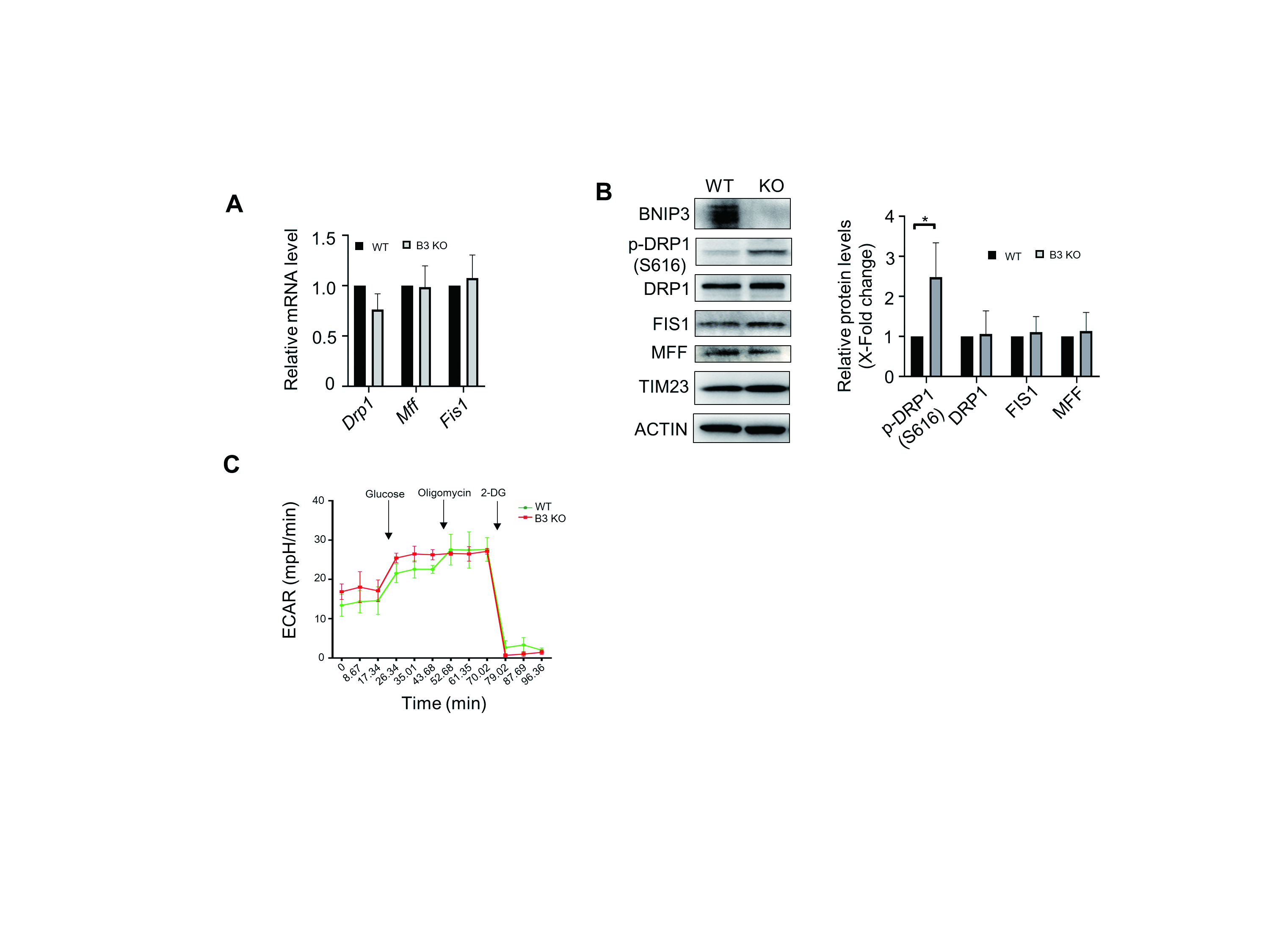

Supplement: Supplementary file 4 — Supplementary Figure 3 [file 41419_2022_4795_MOESM4_ESM.tif]

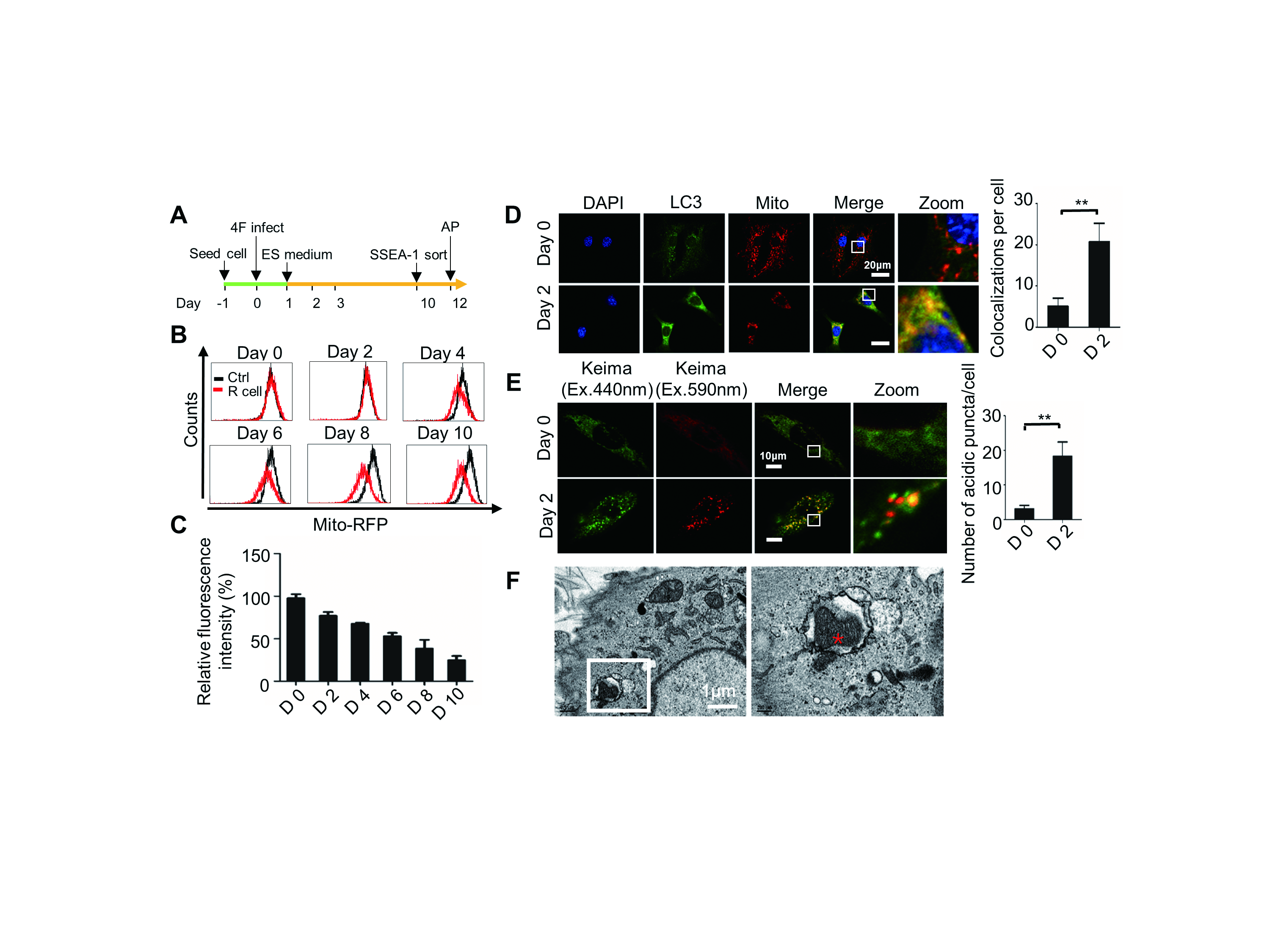

Supplement: Supplementary file 5 — Supplementary Figure 4 [file 41419_2022_4795_MOESM5_ESM.tif]

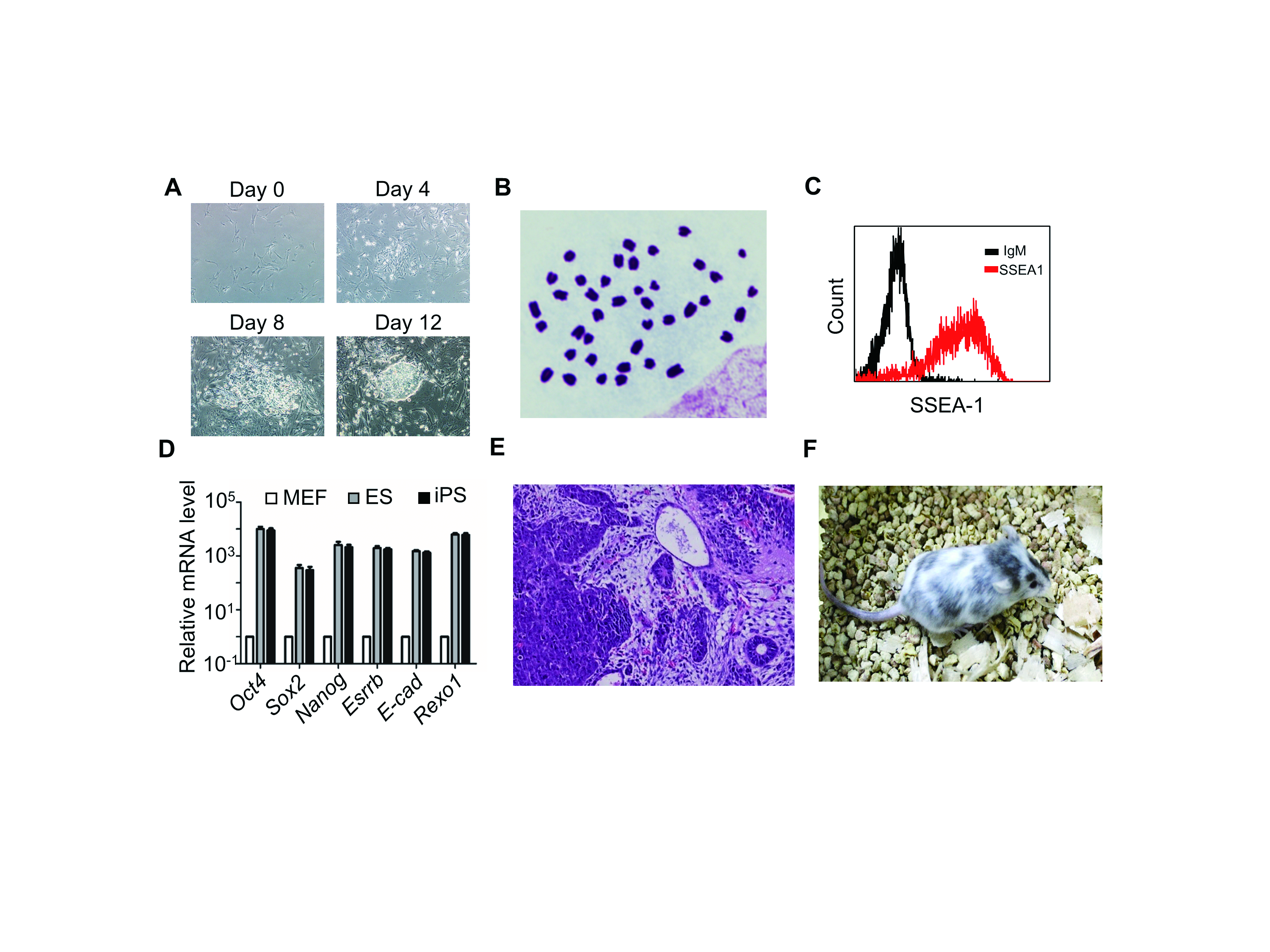

Supplement: Supplementary file 6 — Supplementary Figure 5 [file 41419_2022_4795_MOESM6_ESM.tif]

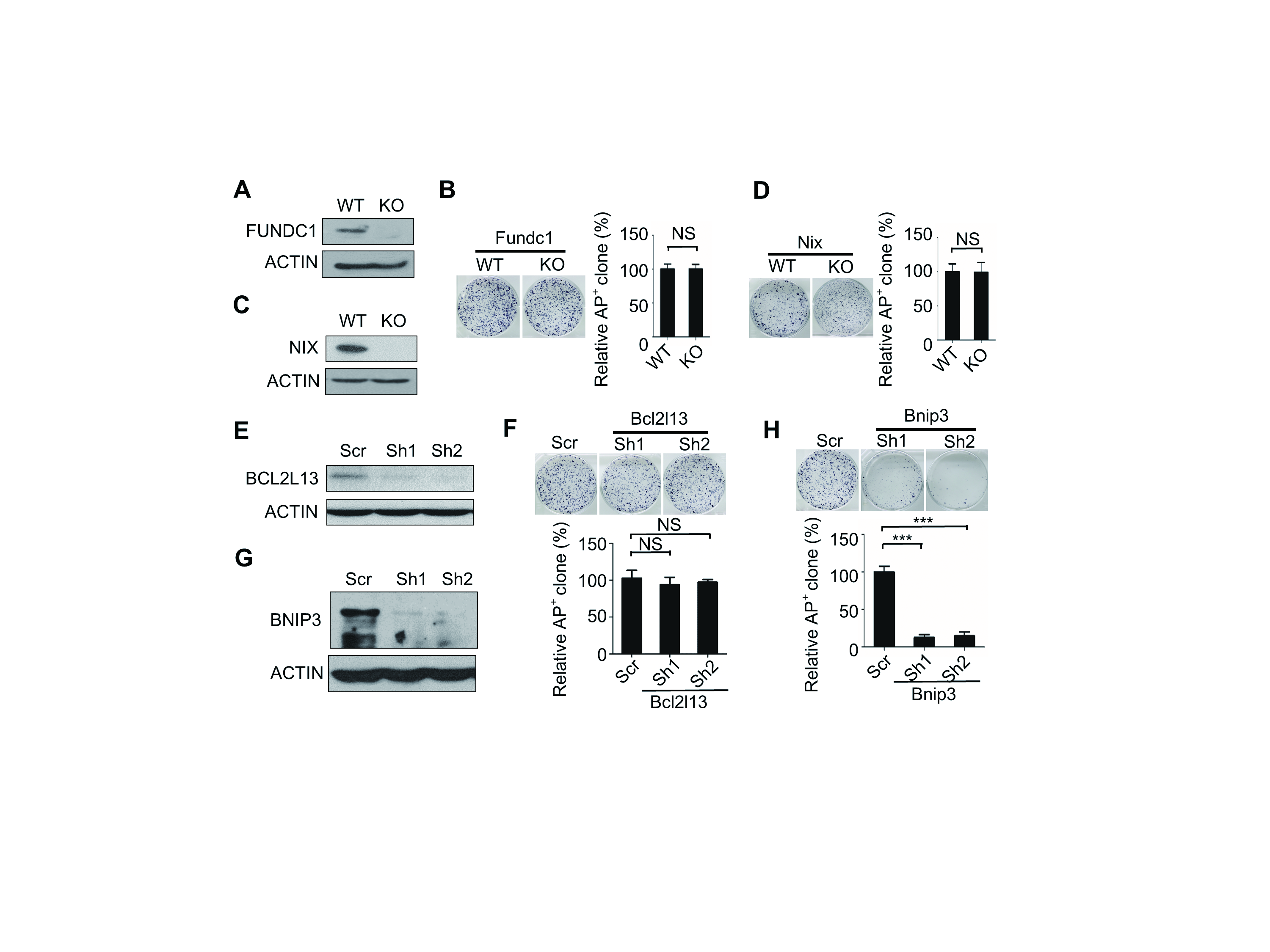

Supplement: Supplementary file 7 — Supplementary Figure 6 [file 41419_2022_4795_MOESM7_ESM.tif]

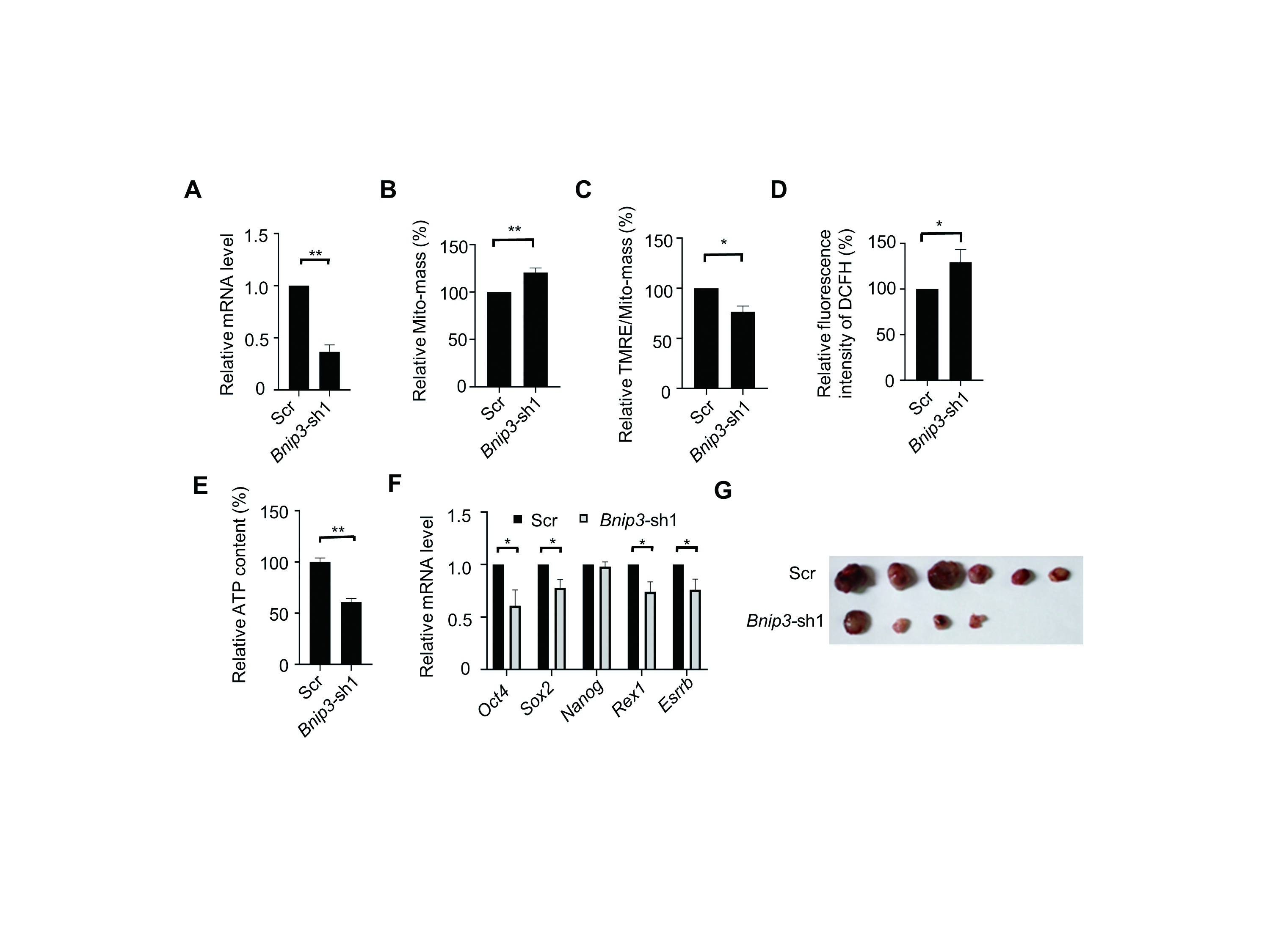

Supplement: Supplementary file 8 — Supplementary Figure 7 [file 41419_2022_4795_MOESM8_ESM.tif]
